# Precision medicine for all? Challenges and opportunities for a precision medicine approach to critical illness

**DOI:** 10.1186/s13054-017-1836-5

**Published:** 2017-10-18

**Authors:** Christopher W. Seymour, Hernando Gomez, Chung-Chou H. Chang, Gilles Clermont, John A. Kellum, Jason Kennedy, Sachin Yende, Derek C. Angus

**Affiliations:** 1 The Clinical Research, Investigation, and Systems Modeling of Acute illness (CRISMA) Center, Department of Critical Care Medicine, University of Pittsburgh School of Medicine, Pittsburgh, PA USA; 2 Department of Medicine, School of Medicine, University of Pittsburgh, Pittsburgh, PA USA; 3 Department of Biostatistics, Graduate School of Public Health, University of Pittsburgh, Pittsburgh, PA USA; 4 Center for Health Equity Research and Promotion, VA Pittsburgh Healthcare System, Pittsburgh, PA USA; 5 Department of Critical Care Medicine, University of Pittsburgh School of Medicine, 3550 Terrace Street, 639 Scaife Hall, Pittsburgh, PA 15261 USA

**Keywords:** Precision medicine, Critical illness, Phenotypes

## Abstract

All of medicine aspires to be precise, where a greater understanding of individual data will lead to personalized treatment and improved outcomes. Prompted by specific examples in oncology, the field of critical care may be tempted to envision that complex, acute syndromes could bend to a similar reductionist philosophy—where single mutations could identify and target our critically ill patients for treatment. However, precision medicine faces many challenges in critical care. These include confusion about terminology, uncertainty about how to divide patients into discrete groups, the challenges of multi-morbidity, scale, and the need for timely interventions. This review addresses these challenges and provides a translational roadmap spanning preclinical work to identify putative treatment targets, novel designs for clinical trials, and the integration of the electronic health record to implement precision critical care for all.

## Background

Recent advances in our understanding of the complex interplay of health and disease have spurred a movement entitled “precision medicine” [1]. Although medicine has arguably always been intended to be precise, this term is generally used to refer to the aspirational goal of demarcating and treating highly specific biologic alterations, such as specific aberrations in gene structure or regulation, transcription, or post-transcription molecular pathways. Thus, patients who present with similar signs or symptoms, or who have, for example, a histologically similar tumor, could be parsed into subsets who have different yet highly specific molecular defects requiring individual treatments.

The promise of precision medicine, therefore, is that a greater understanding of individual data will lead to personalized treatment and improved outcomes. Optimism for this approach arises from examples like the targeting of the single gene mutation in the human epidermal growth factor receptor 2 (HER2) gene in breast cancer cells with a monoclonal antibody, Trastuzumab [2]. The subsequent completion of the Human Genome Project [3], molecular subtyping of melanoma on *BRAF*, *RAS*, and *NF1* mutations [4], and multiple other breakthroughs offer additional hope for a personalized future. However, while such examples are spectacular, the challenge today is moving precision medicine to our most common conditions. In this review, we discuss the evolution of precision medicine with a focus on its application to the field of critical care.

**Fig. 1:**
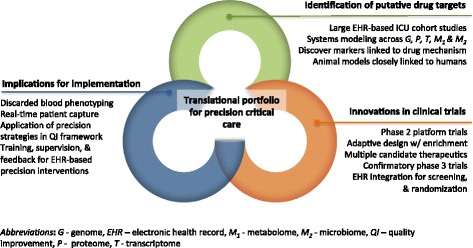
Roadmap for a portfolio of precision medicine in critical care, including integration of preclinical studies, translational work, clinical trials, and implementation science

We begin by discussing salient terms and concepts. We next address major barriers to precision medicine that, although not unique to critical care, are relevant to our future agenda, including how to appropriately select groups of patients for specific treatments; incorporate the complexities of multi-morbidity; conduct studies of adequate scope and scale; and generate data that are adequately timely. Finally, we discuss a road map to achieve this vision (Fig. 1), from preclinical and translational work to novel clinical trials and ultimately integrating into current practice with implementation science.

## Terminology

The broad lexicon of “precision” medicine includes many overlapping and somewhat confusing terms (Table 1). Initially, the terms “individualized medicine” and “personalized medicine” were used. However, as researchers sought specific biologic pathways, they recognized that such pathways might apply to a group of patients, and not necessarily to a single or unique individual. Hence, at least in the near future, the goal is not necessarily to generate a unique treatment for every individual or person, but, rather, tailored treatments for groups with tightly grouped biologic features. These groups, in turn, need a name.

**Table 1:** Key terms in precision medicine

Key term	Description
General	Individualized—Treatments are unique to each individual Personalized—Treatments prioritize patient needs and preferences Precision—Treatments optimized for genetics, lifestyle, and environment Stratified—Setting of patient groups into narrow “strata” by tightly grouped biological features
Grouping	Stratum—Tight groupings of patients defined by similar sets of biological features Phenotype—Clinical features or traits that characterize a group of patients within a disease or syndrome, including genetics, environmental factors, and other clinically observed characteristics Group—A portion of a patient population within a larger group showing decreased inter-subject variability and/or different prognosis and behavior of disease from the larger disease population
Sub-grouping	Endotype—Biological subtypes defined by distinct pathophysiologic mechanisms within a phenotype Subphenotype—Similar to an endotype, but without necessarily showing mechanism or causality Subtype—A broader term for the division of a patient population by any observable characteristics
Phenotype categories	Prognostic—Indicators used to inform about risks of various outcomes Predictive—Indicators providing information about the likelihood of response to a given treatment Drug response—Differential responses to drug based on phenotype defined by an indicator Device response—Differential responses to device based on phenotype defined by an indicator
Heterogeneity of treatment effects (HTE) and enrichment	HTE—Differences in treatment responses in a group due to variability in drug response phenotype within that group Enrichment—A prospective strategy for addressing HTE by reducing heterogeneity of the sample population or increasing representation of patients with similar risk profiles

### Strata, phenotypes, endotypes, subphenotypes, and subtypes

Some use the term “stratified” medicine instead of precision medicine, and advance the idea that groups are “strata” [5]. Others borrow from genomic medicine and use the term “phenotype” to describe a group or cluster of patients, albeit more broadly than in its original framing. The original use of phenotype would include the entire clinical expression of a condition, including its trajectory and outcome. For example, the phenotype for some severe genetic mutations is intrauterine death. But phenotype in the lexicon of precision medicine can be used quite differently. For example, a clinical trialist typically uses the term phenotype to describe a set of presenting features that could be used as criteria for enrollment into an experimental study. In this example, the subsequent trajectory and outcome are not part of the definition of phenotype. Neither use is wrong, but it is key that researchers are explicit in delineating whether phenotype is restricted to presenting features, and if so at what time point, or not.

If a group of patients look similar (share a presenting phenotype) but have subclinical differences (e.g., two different gene expression patterns or metabolic profiles), then they might be further divided into “endotypes”. Thus, a particular phenotype may be expressed by two endotypes. However, endotype in its strictest sense implies an understanding of the exact mechanism of disease, or at least the mechanistic relationship between the molecular signature defining the endotype and the disease in question [6]. In reality, researchers may often be able to associate particular patterns of gene expression or biomarker profiles with disease characteristics, but fall short of proof of mechanism and causality. Thus, the term “endotype” may be used rather loosely, prompting others to prefer the term “subphenotype” (or “subtype”).

A problem with “subphenotype” is that it too is used in multiple ways [7]. One usage implies that “sub” means “under the surface”, and thus refers to a grouping of patients by characteristics not typically visible clinically. This definition is a hedge on endotype, recognizing that the subclinical features may help divide patients, but do not necessarily explain or cause disease events. The second use of “subphenotype” uses “sub” to mean division, like dividing a set into subsets, and is applied to divide up clinically similar groups by further division of their clinical features. Thus, we could divide the phenotype “acute respiratory distress syndrome (ARDS)” into subphenotypes in two different ways. First, we may say that we find subphenotypes based on differences in biomarker patterns (where “sub” means “under the clinical surface”) [8]. Alternatively, we may find different subphenotypes based on differences in presenting clinical features (e.g., extrapulmonary versus pulmonary ARDS [9]. In practice, researchers are sometimes unclear on which nomenclature they are applying and blend the two approaches. Finally, others simply use the term “subtype”, which avoids any specific clinical or mechanistic inference [10].

### Prognostic, predictive, or drug (device)-responsive characteristics

The manner in which we describe groupings is also important and requires an understanding of three common terms: prognostic, predictive, and drug-responsive (or device-responsive). These terms relate to the clinical implications of any particular group, phenotype, set of patients or set of markers for such groups. “Prognostic” means “predictive of, or associated with, a subsequent clinical event or outcome”. What is not always clear is whether the term is applied to the outcome regardless of whether it is caused by the disease of interest. For example, a biomarker could be described as “prognostic” if it identifies a set of patients with acute myocardial infarction (AMI) at a greater risk of death, regardless of whether the death is due to the AMI (so-called “cardiac-related death”) or not. “Predictive” resembles prognostic but is in fact synonymous with “drug-responsiveness” (or device-responsiveness) and is a term used by groups such as the Food and Drug Administration (FDA) to specifically imply an approach to divide patients based on their likelihood of responding to a drug or device [11]. Thus, one marker may be prognostic, in that it predicts the likelihood of having the outcome of interest (e.g., death). Another marker may be predictive (or drug- or device-responsive), in that it predicts whether a patient’s risk of the outcome, such as death, will *change* when given a particular drug or device.

Any particular marker may be prognostic only, predictive only, prognostic and predictive, or neither. For example, in ARDS, severe hypoxia may be both prognostic (associated with greater odds of death) and predictive (associated with greater likelihood of benefit from proning). Older age may also be prognostic, but not predictive of benefit from proning. When treating a deep venous thrombosis, a cytochrome P450 mutation may have no impact on the natural history of subsequent thrombotic events (not prognostic) yet be exquisitely predictive of whether a given dose of warfarin is capable of changing the risk of subsequent thrombosis (predictive) [12, 13]. The labeling of markers or subgroups of patients as prognostic or predictive is further complicated because their properties may be putative, unknown or unanticipated, and researchers may apply the terms loosely.

Groupings or markers that have “predictive/drug-responsiveness” characteristics, either known or putative, can be thought of in three complementary ways during research. First, retrospective studies can uncover treatment by subgroup interactions from existing data, which may correspond to different drug-response phenotypes. These are only associations. Alternatively, drug response characteristics can guide enrollment criteria into clinical trials (so-called “enrichment” strategies), with the goal of ensuring that only patients most likely to respond to a therapy are enrolled in the trial [14]. Finally, they can be used after-the-fact to help parse out within a trial the way in which a drug appeared to work better in some patients and not in others—so-called “heterogeneity-of-treatment-effect” (HTE) [15]. Ideally, an HTE analysis of a broadly enrolling trial could provide clues to potential biomarkers or phenotypes of patients that are predictive of drug response. The final approach is the closest to generating causal inference with respect to treatment by subgroup interactions, although the statistical tests that result in trial conclusions typically speak to the likelihood of groups having different effects than the magnitude of the observed differences.

## Reconciling precision medicine with current practice

Many rightly argue that medicine has always attempted to be precise. Clinicians and researchers always seek to make a specific diagnosis, converting where possible vague signs and symptoms into specific diseases and, when that is not possible, at least into something close to a disease—a “syndrome”. Such efforts seek to group patients based on anticipated actions, such as whether to give a particular treatment, or whether to forecast a likely prognosis. For example, a “diagnostic” biomarker, such as a troponin for AMI, is assumed to be inherently valuable in the effort to establish the diagnosis of acute myocardial infarction. Yet, the value of having a diagnosis is simply the sum of the value gained through determining and initiating a treatment plan, stopping the search for other diagnoses (and treatment plans), and offering counsel to the patient and family on prognosis.

Thus, under the traditional medicine rubric, a diagnostic marker is assumed to be valuable because we assume it is valuable to make a diagnosis. It is worth remembering, however, that the value of making a diagnosis is the sum of both the value gained in prognostic and predictive/drug-responsive accuracy, each a core element of precision medicine. Put another way, precision medicine is simply ensuring that “diagnostic” accuracy is not of given value in its own right, but rather tied to explicit gains in accuracy for tailored treatments and provision of prognostic information.

Another important characteristic of current best practice is the use of protocols. Some may view the increasing use of protocols as contrary to precision medicine. A protocol is intended to ensure that the practitioner does the same thing each time he or she is faced with a particular situation. Yet, protocols and precision medicine are likely complementary. For example, consider the following protocol instruction: when systolic blood pressure < 90 mmHg, if CVP is < 8 cm H_2_O, then give fluid, otherwise increase vasopressor. This instruction is in fact being “precise”, tailoring the choice of fluid or vasopressor for hypotension based on additional “individual” considerations. At the same time, while also attempting to ensure precise management, it is standardizing delivery via the use of explicit instructions. In the case of critical care, our level of evidence does not commonly support the use of precision strategies in clinical practice guidelines or protocols—*yet*. For example, the most recent Surviving Sepsis Campaign guidelines have few if any precision strategies for diagnosis or treatment included [16]. But this does not mean that the two are mutually exclusive approaches to care. Indeed, many argue that the implementation of complex precision medicine strategies will require a protocolized approach [17].

## Four challenges to the implementation of precision medicine in critical care

### 1. Finding discrete groups and subgroups of patients

We state above that traditional medicine has always sought to divide patients into groups, but this is also a key element for precision medicine. What is often overlooked in both is that assigning a patient to a particular group is non-trivial. Clinicians are long familiar with diagnostic challenges, differential diagnoses and vague syndromic presentations. Regrettably, that challenge is not easily solved by precision medicine. Although early “wins” in precision medicine rest on very unique and particular genetic aberrations that appear to account for the entire spectrum of clinical consequences, we would be naïve to think that such explicit and deterministic answers underlie all expression of disease. Instead, for some time to come, all efforts in precision medicine will still have to embrace several wide, long-standing, and potentially uncomfortable, domains of uncertainty.

Three types of uncertainty commonly arise when attempting to divide patients into discrete groups: missingness, continuity, and multiplicity. Missingness is a common problem when we fail to measure what is needed, either because we do not know what to measure, or we are unable to do so. Second, even if we have measured an important characteristic, it may be expressed on a continuum, yet it has to trigger a discrete action: give or not give therapy x. Thus, we need to determine the breakpoint on the distribution. Third, the problem of interest may have multiple domains or dimensions. Having more dimensions suggests we may better understand complex problems, but the data across the different dimensions may be hard to reconcile and shrink to guide discrete decisions.

Thus, consider, for example, that we wish to block an immune checkpoint pathway, such as programmed death 1 (PD1). If we wish to select a patient to administer anti-PD1, we may trip up over problem 1—missingness—not knowing or having a rapidly deployable assay or biomarker of PD1 expression. Alternatively, we may have an assay, but PD1 expression may be very common and variable in sepsis, and we may struggle to know “how much expression” makes a patient a good versus a bad candidate for suppression of that expression—problem 2. Finally, we may appreciate that the potential benefits of an anti-PD1 strategy may be more complex and depend on the measurement of several characteristics of the host’s immune status, as well as characteristics of the invading pathogen. When measuring these characteristics, we may yield potentially complicated and conflicting information: some measures of immune status suggest suppression would be good and some suggest it would be bad—problem 3. These issues are further complicated by different statistical methods to group patients that may not always return the same discrete groups.

### 2. Incorporating multi-morbidity

Researchers and clinicians are increasingly recognizing that patients encountering the health care system do so not with an isolated problem on a background of good health, but with potentially one new problem on a background of multiple pre-existing comorbidities. The combination of these comorbidities adds considerable complexity and has been termed “multi-morbidity” [18].

In critical care, patients also present with multi-morbidity, such as hypertension, diabetes mellitus, and chronic obstructive pulmonary disease, but now complicated by one or many acute organ dysfunctions. More than 60% of the US population over the age of 65 years has two or more comorbid conditions [18, 19]. And among those admitted to the ICU, patients multi-morbidity account for one in three. Multi-morbid patients generate high costs, incur more adverse events, and for a given critical illness, experience a worse outcome compared to patients without comorbidity [20]. And, pre-existing chronic disease may also influence the recovery from critical illness. Therefore, the outcomes of these patients not only vary based on which pathways are activated during the critical illness, but also on the interaction between these pathways and chronic diseases.

Despite the burden of multi-morbidity, our knowledge about multi-morbid patients is not congruent with how often we care for them. For example, while patients with chronic disease are regularly included in randomized trials, many multi-morbid patients at the more severe end of the spectrum will be typically excluded using criteria like “underlying disease with poor prognosis.” In the EDEN trial [21], among 6968 screened but excluded patients, approximately 3300 (47%) had some form of chronic disease for which they were not enrolled.

**Fig. 2:**
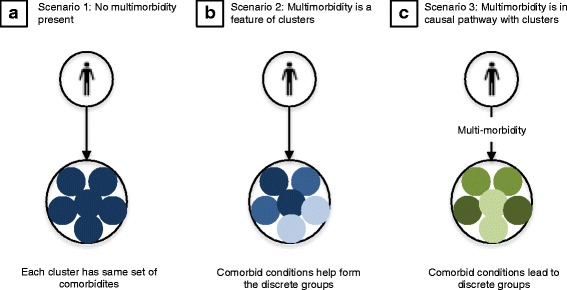
Examples of different ways in which multi-morbidity can contribute to clusters in precision medicine. In **a**, all *dark blue clusters* have similar co-morbidity patterns, while *multiple blue colors* represent clusters in which co-morbidity is a contributing feature (**b**). In **c**, multi-morbidity is in the causal pathway to various clusters, but itself is not a defining feature

Multi-morbidity may also challenge the development of precision critical care (Fig. 2). First, multi-morbidity may be present and measurable, but unrelated to the discrete clusters that may guide precision treatment. Second, multi-morbidity could itself be part of the set of variables evaluated when identifying discrete groups. In chronic obstructive pulmonary disease (COPD) phenotyping using principal components analysis, [22] the presence of obesity, cardiovascular comorbidities and diabetes were included in the hierarchical clustering algorithm and ultimately found to be key features in a subgroup with a higher risk of mortality but less severe airflow limitation.

Finally, multi-morbidity could be investigated as a causal factor in phenotypic variation, perhaps through changes in the epigenome. The epigenome refers to chemical modifications of DNA and DNA-associated proteins that regulate gene expression [23]. Common epigenetic changes include DNA methylation or histone modification, and occur as natural process influenced by the environment or disease. The result is that certain genes are turned “on or off” in various cells in sometimes random fashion. Evidence for the epigenetic contribution to disease can be found in twin concordance studies, where heritable features in various cancers were present in a variable proportion of pairs [24]. Thus, most of the variation in sporadic cancer could be attributed to environmental factors and somatic events. Such phenomena could be generally hypothesized in the immunosuppressive features of critical illness, where the epigenome may be important in regulation of immunologic pathways, as reported during monocyte differentiation in response to lipopolysaccharide [25].

### 3. Managing the scale of clinical and biologic data

**Fig. 3:**
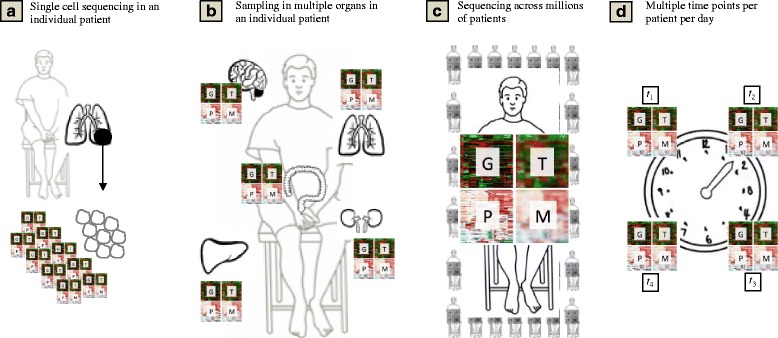
Challenges of scale in precision medicine. **a** How single cell sequencing in a sample from an individual generates thousands of data points. **b** How multiple organs within a patient can be sampled, while **c** studies may now enroll millions of patients. **d** Complexity and volume of data for precision medicine dramatically increases when individuals are sampled over multiple time points. *G* genomic data, *T* transcriptomic data, *P* proteomic data, *M* metabolomics or microbiome data

Research in precision medicine is faced with an enormous burden of information (Fig. 3). To develop new scientific knowledge from large-scale studies that explore phenotypes or endotypes, a wealth of data must be gathered, analyzed, and integrated across many levels. Some estimate the volume of genetic sequencing data has grown tenfold each year since 2002 [26], derived from an increasingly variable set of experimental techniques, dimensionality of data, and noise from high-throughput analyses. No longer will these studies use case report forms with 10 to 20 variables in 500 patients measured at a single time point. Instead, any variety of data from genomic, transcriptomic, or sequencing data could be summed from a single cell, further increased by a variety of factors like the number of organs sampled (*O*), subjects c sampled (Eq. 1):

$$ {\displaystyle \begin{array}{c}\hfill Scale\kern0.5em of\kern0.5em data=\kern0.5em \left[\underset{i\kern0.5em =\kern0.5em 1}{\overset{4}{\varSigma }} Di\right]\kern0.5em \times \kern0.5em O\kern0.5em \times \kern0.5em N\kern0.5em \times \kern0.5em T\hfill \\ {}\hfill \begin{array}{llll} where,\hfill & where,\hfill & where,\hfill & where,\hfill \\ {}{D}_1=\kern0.5em \mathrm{data}\kern0.5em \mathrm{from}\kern0.5em \mathrm{genome}\hfill & O\kern0.5em \in \left\{ 1,\kern0.5em ..o\right\}\hfill & N\kern0.5em \in \left\{ 1,\kern0.5em ..n\right\}\hfill & T\kern0.5em \in \left\{ 1,\kern0.5em ..t\right\}\hfill \\ {}{D}_2=\kern0.5em \mathrm{data}\kern0.5em \mathrm{from}\kern0.5em \mathrm{transcriptome}\hfill & \hfill & \hfill & \hfill \\ {}{D}_3=\kern0.5em \mathrm{data}\kern0.5em \mathrm{from}\kern0.5em \mathrm{proteome}\hfill & \mathrm{if}\kern0.5em o\kern0.5em \mathrm{is}\kern0.5em \mathrm{the}\kern0.5em \mathrm{number}\kern0.5em \mathrm{of}\kern0.5em \mathrm{organs}\hfill & \mathrm{if}\kern0.5em n\kern0.5em \mathrm{is}\kern0.5em \mathrm{the}\kern0.5em \mathrm{number}\kern0.5em \mathrm{of}\kern0.5em \mathrm{patients}\hfill & \mathrm{if}\kern0.5em t\kern0.5em \mathrm{is}\kern0.5em \mathrm{the}\kern0.5em \mathrm{number}\kern0.5em \mathrm{of}\kern0.5em \mathrm{points}\hfill \\ {}{D}_4=\kern0.5em \mathrm{data}\kern0.5em \mathrm{from}\kern0.5em \mathrm{metabolome}\hfill & \mathrm{sampled}\hfill & \mathrm{enrolled}\hfill & \mathrm{sampled}\hfill \end{array}\hfill \end{array}} $$

For example, a hypothetical study which seeks to gather data across a platform of system biology data types could generate more than one million SNPs from single cell genome sequencing, 40,000–50,000 microarray transcripts in transcriptomic analyses, 700 results from proteomic analyses, and 4000–7000 named and unnamed metabolites. Rather than 1000 patients, precision medicine studies have proposed enrollment of over one million patients in whom multiple organs can be sampled at more than one time point [27]. The resulting scale of data, in which to search for important subgroups, would exceed a trillion (2 × 10^12^) data points. Such an increase would directly impact computational time to estimate even simple queries across relational datasets.

There are a variety of potential solutions to the problems of scale in precision critical care. First, computational systems biologists will need to tackle this wealth of data and use state-of-the-art software to coordinate and index omics datasets (e.g., format, store, calibrate). From genome assembly to read mapping to bioinformatics analysis of RNA-sequencing data, immense data storage and sophistication is required for interpretation [26]. Second, we must study how the “interactome”, or protein–protein interactions, and their networks work together [28]. These efforts will require advanced graphical and pathway analysis, and combine genotype, gene expression, and other data to identify dysregulated pathways, infer mechanism, and potentially explain disease heterogeneity. Such efforts rely on further characterization and annotation of unknown molecules that comprise the interactome.

A potentially useful conceptual approach to managing the scale and complexity of omics data is to focus on the search for emergent properties. Emergent properties are thought of as those that cannot be entirely explained by their individual components. Historically, Alexander referred to emergence as high-level causal patterns not directly expressed by fundamental entities or patterns [29], thought to be both irreducible and unpredictable. For example, consciousness or human cognition is a systems level property quite unexpected from individual neurons themselves. And in other fields, emergent properties can explain complex thermodynamics or behavior of birds in a flock. In critical care, complex data resulting from precision medicine research also contain multiple integrated levels, changes over time, and variable environments in the intensive care unit (ICU), and derive from patient subsets often without a gold standard. Findings at the level of a single nucleotide polymorphism or metabolite may be incredibly difficult to parse from trillions of data points, and reveal only a minute part of the underlying disease heterogeneity. Rather, the emergent properties at a macro level could, in a sense, be the broad, common phenotypes for which we search. It may be debated what “macro” is in this context, perhaps a single cohort of patients with thousands of correlated measurements over time in electronic data, or a small cohort of integrated genomic, transcriptomic, and proteomic profiles. Both of these would require computational strategies to uncover patterns in the data leading to otherwise clinically obtuse phenotypes.

An alternative approach is the search for the rare phenotype, in which a single mutation results in a distinct clinical and biologic characteristic among a subset of patients. A classic example is the case of the single gene mutation in the human epidermal growth factor receptor 2 (HER2) gene in breast cancer cells. By investigating carefully in families with a hereditary susceptibility for breast cancer, scientists ultimately located a drug response phenotype for the monoclonal antibody Trastuzumab and an efficient method to identify cases most suitable for treatment. In critical care, a corollary could be the search for susceptibility to meningococcemia either from tumor necrosis factor (TNF) mutation −308 or migration-inhibitory factor (MIF). Although the relationships between these mutations and susceptibility to sepsis and death are not fully unpacked, such mutations would require the key step of combing across millions of potential sepsis cases to locate those for further consideration.

In the end, critical care may solve the immense problem of scale through both system level analyses that search for emergent properties in complex, electronic data and reductionist approaches that isolate single mechanisms with rare phenotypes.

### 4. Obtaining “real-time” data

Many of the notable gains in precision medicine have occurred in oncology, where a patient’s clinical information and biologic samples can be studied for days or months to determine tumor phenotype. Yet in the field of critical care, we face life threatening emergencies like sepsis, shock, or impending respiratory compromise, all of which require prompt treatment to improve outcomes [30]. However, the need for timeliness of care imposes a major barrier to advancing precision treatment, particularly if the technology or assay required to target therapy requires hours or days to complete. For example, in pediatric septic shock, future treatment could be based on patterns of genomic expression. These approaches, although promising and increasingly timely, may still require hours to complete. Such delays could have consequences for the development of organ failure and even mortality if they lead to delays in the emergency department care of pediatric sepsis.

And yet, of all the barriers, timely turn around for phenotyping information may be the most easily solved—particularly by partners in industry. For example, sequencing-based approaches to analyze the microbiome (e.g., 454 pyrosequencing, Illumina MiSeq) required batching of large numbers of specimens and days to weeks of sequencing and informatic analysis. By contrast, using recent advances in nanopore seqencing technology (e.g., Oxford Nanopore), specimens can be individually sequenced and analyzed with interpretable microbiome data available within a single day.

## Roadmap for a translational agenda in precision critical care

To make gains towards precision medicine for all of critical care, a multidisciplinary, collaborative approach is needed that spans three research domains: preclinical work, novel clinical trials, and implementation science (Fig. 1) [17]. Below, we describe a translational roadmap for each domain, using illustrative examples from critical care.

### Pre-clinical science: identifying putative targets for precision therapy

The identification of mechanism-targeted, effective treatments is the first, challenging step in a precision strategy. Amidst the biologic complexity of critical illness, there are large knowledge gaps about the mechanistic determinants of disease that govern a patient’s trajectory. The involvement of multiple interconnected response pathways in critical illness and the effect of treatment creates a difficult path when trying to mimic single-gene targeted treatments found in other diseases. Thus, combinations of many steps are required to build adequate evidence for precision medicine therapies. These include large cohort studies for phenotype discovery work and the identification of candidate targets through integrated systems modeling. Follow-up would include translating clinical and biologic phenotypes into putative biologic mechanisms, modeling these endotypes in animal models closely linked to humans, and in vitro pre-clinical studies of candidate therapies.

For example, preclinical work to identify putative drug targets in sepsis has drawn from the rheumatologic disease paradigm [31]. Clinical studies suggested that subgroups of patients with adequate control of infection may develop unique inflammatory phenotypes in up to one-third of cases: thrombocytopenia-associated multiple organ failure (TAMOF), sequential multiple organ dysfunction (SMOF), immunoparalysis, or macrophage activation syndrome (MAS) [31]. Characterized by biomarkers such as ferritin or thrombocytopenia, these inflammatory phenotypes have distinct pathobiology and specific risk factors and biomarker responses to therapy in vivo. A more definitive translational step was a secondary analysis of a phase III trial of recombinant IL-1 receptor antagonist (anakinra) [32]. In septic patients with criteria similar to macrophage activation syndrome (i.e., disseminated intravascular coagulation and/or hepatobiliary dysfunction)—a syndrome favorably responding to IL-ra blockade in rheumatologic disease, there was a 30% absolute reduction in mortality from treatment vs. placebo (65 to 34%) [33]. This was not seen in patients without MAS features.

Such examples highlight the potential for endotypes to guide precision care in critical illness. But a larger body of multidisciplinary preclinical work is required to understand the basic mechanisms at play, how these mechanisms interact as the disease progresses, and discovery of clinically relevant endotypes for treatment.

### Clinical trials: testing putative targets in clinical populations

To determine if drugs identified in preclinical work are suitable for patients, they must undergo rigorous evaluation in human clinical trials. Yet, critical illness is often described as a graveyard for pharmaceutical trials, in part because multiple “one-population, one-therapy, one disease” trials are neutral or fail to show benefit. In sepsis alone, multiple studies of therapeutics targeting specific molecular mechanisms have failed to find benefit [34, 35]. A number of characteristics of traditional clinical trials make them problematic for future evaluation of precision medicine strategies: i) homogenous populations without complex comorbid conditions; ii) typically a single therapy vs. control group; iii) stopping rules linked to efficacy of a single experimental treatment; and iv) fixed randomization strategy that is typically one case per one control [36, 37].

An alternative approach is to consider the use of novel so-called “adaptive” trial features that are intended to help researchers learn about well-performing drugs and better-targeted subgroups more efficiently. Recently, integrated “platforms” have been developed that seek to take advantage of several design features simultaneously—all of which facilitate the testing of multiple precision medicine approaches [36]. Platform trials can investigate multiple treatments across multiple groups in the same trial, using sophisticated rules for patient, treatment, and site allocation. One feature of a platform trial is the use of a common control arm for multiple comparisons to different experimental therapies. Another is to incorporate response-adaptive randomization rules that preferentially allocate therapies to different phenotypes based on their performance during the trial, thus minimizing the exposure of potentially inferior drugs overall or within trial subpopulations. Another feature is that the trials can span across more than one traditional phase. For example, instead of conducting separate phase 2 and 3 trials, pre-set rules could allow a seamless transition from phase 2 to phase 3. Finally, platform trials, by running continually and perpetually, offer the efficiencies of avoiding downtime in-between trials. The I-SPY2 program in breast cancer is emblematic of these platform designs—and their applicability to precision medicine. I-SPY2 tests multiple treatments in multiple biomarker-defined subgroups, with pre-set rules that promoted efficient learning and preferential treatment assignment [38]. This platform has evaluated more than 12 experimental treatments across eight patient subtypes since beginning enrollment in 2010. For example, the tyrosine kinase inhibitor neratanib was advanced to a phase 3 trial after enrolling less than 200 patients in an adaptive design [39].

In addition to candidate therapeutics, platform trials can also test existing therapies, while leveraging the efficiencies of the electronic health record (EHR). This design is termed REMAP (randomized, embedded, multifactorial, adaptive, platform) [40]. REMAPs can use the EHR to screen for eligible patients, and randomize patients to a variety of candidate therapies. By learning about which patients do or do not have better outcomes with a treatment A vs. B vs. C, the EHR could be used to enrich the eligible cohort for phenotypes more likely to have benefit—so-called response adaptive randomization. The ICU is already a data-rich environment with considerable electronic data capture, even in environments that do not yet have a full EHR. Consequently, a REMAP has already been designed to test optimal strategies for the care of severe pneumonia—REMAP CAP—and has funding through the European Union Platform for European Preparedness Against Re-emerging Epidemics (PREPARE) network and through the Australian and New Zealand governments. In this trial network, multiple treatments are planned for patients hospitalized with severe acute respiratory distress in the ICU. These interventions include various antibiotics, ventilator strategies, and immunomodulation across a variety of patient groups and more than 100 ICUs in Europe.

Taken together, the changing pace of discovery work in precision critical care will mandate that trials of candidate therapies are nimble, accessible, and designed to test multiple therapies across heterogeneous patients.

### Implementation science: moving precision care into ICU practice

Novel strategies to improve critical care outcomes through precision medicine will require close collaboration with experts in implementation science [17]. Some might consider a research program about a precision medicine initiative not even complete until its clinical utility has been rigorously implemented and tested. Second, precision medicine research and clinical care are primed to co-exist, and do so already in many large health care systems such as Kaiser Permanente. These systems coordinate case finding, referral, data measurement, performance metrics, and enrollment in trials—all key steps to generating evidence for precision medicine. And when determined as the new, gold-standard evidence, precision medicine strategies will require methodologists expert in integration in routine practice.

One key to implementation of precision care is feasibility in real-world settings. There are multiple pragmatic steps, including: i) identification of cases who have clinical features of the phenotype/endotype/group of interest; ii) measurement of characteristics that are closely associated with phenotype/endotype/group of interest; and iii) prompting of clinicians to administer mechanism-targeted therapy. All of these steps can be conducted in a “light touch” approach that minimizes contact with team members outside of the bedside care team [41]. For example, the EHR can be leveraged to scan for characteristics derived from vital signs or laboratory results, while discarded blood, urine, and other biologic samples stored in the laboratory can be used to measure prognostic or predictive biomarkers that are not standard of care. These almost real-time interventions would form the basic components for recognition and treatment.

Once established, a precision approach will require a variety of strategies to be durable. These include effective education of the clinical team, consideration of appropriate incentives, and novel quality improvement frameworks to audit and provide feedback. Beyond getting the treatment delivered to the patients most likely to benefit, health systems that invest in electronic health record solutions for measuring the prevalence of the phenotype, and outcome among treated and untreated patients will be better positioned as learning networks. Such data can contribute to large distributed networks across systems and provide stakeholders with more robust information across centers.

## Conclusions

Precision medicine in critical care is a key part of our present and future. However, many challenges limit its application for all patients in the ICU. Complex acute illness among patients with multi-morbidity, integrated systems biology data with daunting scope and scale, and critical illness syndromes that lack gold-standard criteria are just some of the many barriers to newer precision strategies. To move past the failures of molecularly targeted therapeutics, novel trial designs will need to embrace and explore heterogeneity of treatment during phase 2/3 evaluation. Future real-world testing and implementation of precision medicine will also require close partnership with electronic health record systems to reduce cost, improve timeliness of patient screening and treatment, and contribute to broader learning healthcare networks.

## Abbreviations

ARDS: Acute respiratory distress syndrome
CAP: Community acquired pneumonia
COPD: Chronic obstructive pulmonary disease
FDA: Food and Drug Administration
EHR: Electronic health record
HER2: Human epidermal growth factor receptor 2
HTE: Heterogeneity-of-treatment-effect
ICU: Intensive care unit
MAS: Macrophage activation syndrome
PD1: Programmed death 1
PREPARE: Platform for European Preparedness Against Re-emerging Epidemics
REMAP: Randomized, embedded, multifactorial, adaptive, platform
SMOF: Sequential multiple organ dysfunction
SNP: Single-nucleotide polymorphism
TAMOF: Thrombocytopenia-associated multiple organ failure.
